# The Polyphenols (−)-Epigallocatechin-3-Gallate and Luteolin Synergistically Inhibit TGF-β-Induced Myofibroblast Phenotypes through RhoA and ERK Inhibition

**DOI:** 10.1371/journal.pone.0109208

**Published:** 2014-10-01

**Authors:** Alana L. Gray, Charles A. Stephens, Rebecca L. H. Bigelow, David T. Coleman, James A. Cardelli

**Affiliations:** Louisiana State University Health Sciences Center – Shreveport, Shreveport, Louisiana, United States of America; Winship Cancer Institute of Emory University, United States of America

## Abstract

The presence of reactive stroma, predominantly composed of myofibroblasts, is directly associated with and drives prostate cancer progression. We have previously shown that (−)-Epigallocatechin-3-gallate (EGCG), in the form of Polyphenon E, significantly decreases serum levels of HGF and VEGF in prostate cancer patients. Given that HGF and VEGF are secreted from surrounding tumor myofibroblasts, these observations suggested that EGCG may inhibit prostate cancer-associated myofibroblast differentiation. Herein, we demonstrate that micromolar combinations of EGCG and a second polyphenol, luteolin, synergistically inhibit TGF-β-induced myofibroblast phenotypes in prostate fibroblast cell lines, as observed primarily by potentiation of fibronectin expression. Functionally, EGCG and luteolin inhibited TGF-β-induced extracellular matrix contraction, an enhancer of tumor cell invasion. EGCG and luteolin inhibited downstream TGF-β-induced signaling, including activation of ERK and AKT, respectively, but mechanistically, only ERK appeared to be necessary for TGF-β-induced fibronectin expression. Furthermore, neither EGCG nor luteolin affected Smad signaling or nuclear translocation. Rho signaling was found to be necessary for TGF-β-induced fibronectin expression and EGCG and luteolin each reduced RhoA activation. Finally, EGCG and luteolin were shown to reverse TGF-β-induced fibronectin expression, implicating that these natural compounds may be useful not only in preventing but also in treating already activated myofibroblasts and the diseases they cause, including cancer. The ability of EGCG and luteolin to synergistically target myofibroblasts suggests that combined clinical use of these compounds could prevent or reverse cancer progression through targeting the tumor microenvironment, in addition to the tumor itself.

## Introduction

Prostate cancer is the most diagnosed cancer in the United States [1]. Although a neoplasia of glandular epithelia, it has become appreciated that prostate cancer progression is not solely dependent on the epithelial compartment, but rather results from coordinated interactions between epithelial cells and the surrounding tumor microenvironment. This microenvironment consists of endothelial cells, immunological cells, stromal macrophages and myofibroblasts [2], [3]. Myofibroblasts are differentiated, non-transformed fibroblasts that are phenotypically characterized by ACTA2 (alpha-smooth muscle actin; α-SMA) expression and secretion of extracellular matrix (ECM) components, including COL1A1 (collagen) and FN1 (fibronectin) [4], [5]. Several cytokines secreted from epithelial cells, including TGFβ1 (TGF-β) and IL-6, are capable of stimulating the reversible conversion of fibroblasts to myofibroblasts [4], [6].

Cancer-associated fibroblasts (CAFs) contribute to the dysregulated wound-healing-like properties of the reactive stroma [7]. CAFs can arise from various cell types within the stroma and populations of CAFs can contain activated (myofibroblasts) and non-activated fibroblasts. Myofibroblasts secrete high levels of growth factors, such as hepatocyte growth factor (HGF) and vascular endothelial growth factor (VEGF), which can bind to cognate receptors on tumor epithelial cells stimulating proliferation, migration, and invasion [5], [8]. It has been shown that reactive stroma is associated with a poor prognosis for cancer patients [9], [10]. Of particular relevance, depletion of various stromal components, including myofibroblasts, can restore normal lymphatic and vascular function in mice with solid tumors, thereby reducing tumor progression [2], [11]. These observations suggest that agents which target myofibroblasts in tumor microenvironments may be clinically useful.

Multiple epidemiological studies have found an inverse correlation between plant-based food consumption and cancer risk [12], and recent emphasis has been placed on the potential use of natural products as preventative or adjuvant cancer therapy. One particular class of compounds that has garnered much attention for potential pharmaceutical use is the flavonoid family. Flavonoids consist of subgroups, such as flavones, flavonols, and flavanols, categorized by structure. Many studies have been conducted examining the effects of flavonoids on tumors; however, relatively few studies have focused on the effects of these compounds on fibroblasts. Of particular interest, there are some publications citing observations regarding flavonoids and fibroblasts. For example, various flavonoids have been shown to reduce signaling through pathways inducing inflammation [13], increase or decrease collagen expression [14], [15], and prevent myofibroblast transdifferentiation [16], [17].

(−)-Epigallocatechin-3-gallate (EGCG) is a flavanol derived from green tea leaves that has been implicated for use in prostate and other types of cancers [15]–[20]. Another natural compound, luteolin, is a flavonoid that is derived from cruciform vegetables that has also been studied in regards to its anti-cancer activities [21]–[23]. EGCG and luteolin have been shown to inhibit numerous cell signaling pathways including platelet-derived growth factor (PDGF), epidermal growth factor (EGF), and HGF signaling axes [18], [24], [25]. Aside from targeting specific cancer-promoting signaling cascades, the anti-cancer mechanisms by which EGCG and luteolin are proposed to function are wide-ranging and include functioning as anti-oxidants, binding to and inhibiting enzymatic activity directly, and altering membrane fluidity [25], [26]. EGCG and luteolin have predominantly been shown to inhibit epithelial cell proliferation, invasion, and tumor growth in xenograft models [18], [20], [23]; however, there remains a gap in our understanding of how these compounds act on cells of the tumor microenvironment, thereby warranting further investigation.

We have previously shown that the plant polyphenols EGCG and luteolin are able to inhibit breast and prostate carcinoma cell motility and invasion stimulated by the growth factor, HGF, *in vitro* [22], [24], [26]. Furthermore, we have published data from a Phase II clinical trial demonstrating that EGCG, in the form of Polyphenon E, is able to decrease serum levels of prostate serum antigen (PSA), HGF and VEGF in men with prostate cancer [27]. These results were recapitulated *in vitro* wherein treatment of the WPMY-1 prostate fibroblast cell line with physiologically relevant concentrations of EGCG significantly decreased secreted levels of HGF and VEGF in conditioned medium. These observations suggested that EGCG can not only target epithelial cells, but also surrounding myofibroblasts.

Herein, we assessed the ability of the polyphenols EGCG and luteolin to inhibit myofibroblast phenotypes stimulated by TGF-β. Specifically, we wanted to determine if combining EGCG with another plant-based compound, luteolin, would result in increased efficacy in targeting myofibroblast phenotypes *in vitro*. We found combinations of EGCG and luteolin that were synergistic in reducing fibronectin expression, a characteristic marker of myofibroblasts. These synergistic combinations also reduced matrix contraction, a key functional consequence of myofibroblast activation. Our data suggest that these compounds work through similar and distinct mechanisms to target myofibroblast activation, and each of these polyphenols was found to reverse myofibroblast activation, as well. Overall, our investigation suggested that EGCG and luteolin alone are able to target myofibroblasts, but combinations of these plant-based compounds result in increased efficacy. Therefore, in combination with previous work, these data implicate EGCG and luteolin as potential therapeutic agents targeting, both, epithelial tumor cells and surrounding myofibroblasts. Theoretically, clinical trial results showing EGCG efficacy in prostate cancer patients could be further enhanced by combining EGCG with luteolin *in vivo*.

## Materials and Methods

### Cell culture

The WPMY-1 prostate fibroblast cell line was obtained from ATCC (CRL-2854) and grown in DMEM, 5% FBS, 25 µg/ml penicillin-streptomycin. WPMY-1 cells were derived from the stroma adjacent to a normal adult prostate. These cells were authenticated by Promega through short tandem repeat analysis during the course of experiments. WPMY-1 RhoA knockdown (KD) and non-target shRNA cells were maintained in complete media containing 1.8 µg/ml puromycin. HPS-19I cells [28], [29] were a gift from Dr. David Rowley (Baylor College of Medicine) and were recently analyzed by spectral karyotyping. These cells were maintained in DMEM with 5% FBS, 5% Nu-Serum, 5 µg/ml insulin, 0.5 µg/ml testosterone, 25 µg/ml penicillin-streptomycin.

### Materials

EGCG, luteolin, GGTI-2133, and TGF-β recombinant protein were obtained from Sigma Aldrich. Other materials include U0126 (Calbiochem) and LY294002 (Enzo Life Sciences, Inc.).

### Proliferation and Viability Assays

WPMY-1 fibroblasts were plated at ∼20% confluency and allowed to grow in complete media 1 day prior to treatment with 1.3–40 µM EGCG or luteolin for 24 hours in 1% FBS DMEM or 4 days in 5% FBS DMEM. Data were acquired using an IncuCyte Zoom imaging system (Essen Bioscience). Proliferation data are shown as percent confluency at the indicated timepoints normalized to confluency immediately following initial treatment (T0) to account for any differences in starting confluency. The assay was repeated twice and performed with 8 samples per treatment. Cell viability was assessed following proliferation analysis using the Cell Titer Blue Cell Viability assay (Promega) according to manufacturer instructions.

### Western blot analysis

Fibroblasts were plated at 70% confluency. The following day, the cells were starved in 1% FBS DMEM 5 hours prior to treatment in 1% FBS DMEM with 5 ng/ml TGF-β +/−1.3–40 µM EGCG, luteolin, or the indicated inhibitors for 24 hours. For the C3 transferase experiment, WPMY-1 cells were pretreated with C3 transferase (Cytoskeleton, Inc.) for 8 hours before TGF-β addition for 24 hours. Protein lysates were harvested with boiling laemmli (125 mM Tris; 4% SDS; 0.01% bromophenol blue; 30% sucrose) containing 0.5% β-mercaptoethanol. Primary antibodies were used at 1∶500–5000 at 4°C overnight and include alpha-smooth muscle actin (A5228, clone 1A4, mouse monoclonal) (Sigma); Fibronectin (sc-9068, clone H-300, rabbit polyclonal), Collagen 1A1 (sc-28657, clone H-197, rabbit polyclonal), AKT1/2 (sc-1619, clone N-19, goat polyclonal) (Santa Cruz Biotechnology); Tubulin α-Ab-2 (MS581P, clone DM1A, mouse monoclonal) (Thermo Scientific); Phospho-PTK2 (FAK) (3281, Tyr576/577, rabbit polyclonal), Phospho-AKT (3787, Ser473, rabbit monoclonal), Phospho-SMAD2 (3108, clone 138D4, Ser465/467, rabbit monoclonal), Phospho-p44/42 MAPK (ERK) (9106, clone E10, Thr202/204, mouse monoclonal), TGF-βRI (3712, rabbit polyclonal) (Cell Signaling Technologies, Beverly, MA); TGF-βRII (ab61213, rabbit polyclonal) (Abcam); RhoA (ARH03, mouse monoclonal) (Cytoskeleton, Inc.). Horse radish peroxidase-conjugated secondary antibodies were used at 1∶5000–10000 for 1–2 hrs at room temperature prior to ECL-detection. Images were adjusted for contrast and cropped in Adobe Photoshop 7.0.

### CompuSyn analysis

Synergistic interactions were determined using CompuSyn software (ComboSyn, Inc.). CompuSyn calculates the combination index (CI) which is based on mass-action law. Briefly, concentration ranges of luteolin and/or EGCG were tested for the ability to reduce fibronectin expression by western blot. Images were quantitated by densitometry as measured by ImageJ (NIH). Single dose response curves were entered into CompuSyn as a basis for determining synergy. Combination effects were entered and the combination index of each combination was determined using the equation: [(D1/Dx1)+(D2/Dx2)], such that Dx1 is the dose of Drug 1 that inhibits fibronectin at x% and Dx2 is the dose of Drug 2 that inhibits fibronectin at x% and D1 is the portion of Drug 1 that also inhibits fibronectin at x% in combination with Drug 2 and vice versa. Combination indices are interpreted such that a CI >1 = antagonism, CI = 1 = additivity, CI <1 = synergy [30]. Combination data are presented using the Chou-Talalay plot which plots the effect of the combinations vs. the combination index.

### Three dimensional ECM remodeling

The methods for this assay were a variation of the assay performed by Calvo *et al.* [31]. Briefly, 5×10^5^ WPMY-1 fibroblasts per well were mixed with a final concentration of 4.6 mg/ml rat tail collagen I and 2.2 mg/ml Matrigel (BD Biosciences). Each well of a 24-well plate was covered with 300 µl of the matrix/cell suspension and allowed to polymerize for 1 hour at 37°C. After 1 hour, matrix/cell suspensions were hydrated with 500 µl of 5% FBS DMEM at 37°C overnight. The gels were released from the tissue culture plastic with a spatula, treated with 5 ng/ml TGF-β +/− the indicated concentrations of EGCG and/or luteolin, and allowed to contract for 4 days. The gels were treated with fresh compounds and media on day 2 post seeding. Images were analyzed using ImageJ software to determine gel area. The assay was performed three times in duplicate.

### DiIC16 staining

WPMY-1 fibroblasts were plated at 50% confluency. The following day, the cells were treated with 5–40 µM EGCG or luteolin +/−5 ng/ml TGF-β for 24 hours. DiIC16 (Molecular Probes, Invitrogen) was added directly to the media at a final concentration of 1 µM and incubated at room temperature for 2 minutes. Cells were fixed with 4% paraformaldehyde, washed and nuclei stained with DAPI. Images were captured on an Olympus BX-50 epifluorescence microscope using MetaMorph software.

### Nuclear Fractionation

WPMY-1 fibroblasts were treated with 5 ng/ml TGF-β +/−40 µM EGCG or 20 µM luteolin for 1 hour. Cells were scraped in 500 µl of Buffer A (10 mM HEPES, 1.5 mM MgCl_2_, 10 mM KCl, 0.5 mM DTT, 0.05% NP40, pH 7.9) and incubated on ice for 10 minutes. Lysates were centrifuged at 4°C at 3000 rpm for 10 minutes. The cytosolic fraction was removed and the pellet washed once with Buffer A. The remaining pellet was resuspended in Buffer B (5 mM HEPES, 1.5 mM MgCl_2_, 0.2 mM EDTA, 0.5 mM DTT, 26% glycerol, 300 mM NaCl, pH 7.9). Lysates were homogenized with 10 strokes through a 22 gauge needle and incubated on ice for 30 minutes prior to centrifugation for 20 minutes at 4°C at 24,000 g. Each lysate was analyzed by western blot.

### RhoA activation assay

WPMY-1 fibroblasts were grown to 80% confluency. Cells were starved in 1% FBS DMEM 24 hours prior to treatment with the indicated concentrations of luteolin or EGCG for 30 minutes prior to the addition of 5 ng/ml TGF-β for an additional 15 minutes. RhoA activation was assessed using the RhoA Pull-down Activation Assay Biochem Kit according to manufacturer instructions (Cytoskeleton, Inc.). Briefly, following treatment cells were washed with ice-cold PBS and scraped off the plates in cell lysis buffer on ice at 4°C. Lysates were centrifuged for 5 minutes at 5,000 g at 4°C. Cleared lysates (1 mg/ml) were incubated with 50 µg Rhotekin-RBD protein beads on a rotisserie rotator for 1 hour at 4°C. Bead pellets were washed twice with wash buffer before the addition of boiling laemmli. Protein concentration was assessed using Pierce 660 nm reagent (Thermo Scientific) and equal amounts of protein were analyzed by western blot.

### Quantitative Reverse Transcriptase-PCR

WPMY-1 cells were allowed to grow to 80% confluency in complete media. Cells were removed with 0.025% EDTA and centrifuged 5 minutes at 1100 rpm. Cell pellets were resuspended in 2 ml Trizol (Life Technologies) and RNA was extracted following the Trizol manufacturer protocol. The SuperScript First-Strand kit (Life Technologies) was used to synthesize cDNA. Semi-quantitative PCR was set-up using RT^2^ SYBR Green Flour FAST Mastermix (Qiagen) and run on a Bio-Rad CFX96 Real-Time PCR Detection System. Data were analyzed using Bio-Rad CFX Manager 3.0 software and are shown as relative fold change. Primers were designed using Integrated DNA Technologies PrimerQuest software. Sequences used to analyze RNA expression include: *RHOA* Forward: 5′-CAGTTCCCAGAGGTGTATG-3′, Reverse: 5′-CCCACAAAGCCAACTCTA-3′; *RHOB* Forward: 5′-CGCTGTAACCTCATCTACTT-3′ Reverse: 5′-CAGTGTTGCCACATTCTTC-3′; *RHOC* Forward: 5′-CTACGTCCCTACTGTCTTTG-3′, Reverse: 5′-CGCAGTCGATCATAGTCTT-3′; *FN1* Forward: 5′-TCGGTGTTGTAAGGTGGAATAG-3′, Reverse: 5′-TCGGTGTTGTAAGGTGGAATAG-3′; *ACTA2* Forward: 5′-GATGGTGGGAATGGGACAAA-3′, Reverse: 5′-GCCATGTTCTATCGGGTACTTC-3′; *COL1A1* Forward: 5′-CAGACTGGCAACCTCAAGAA-3′, Reverse: 5′-CAGTGACGCTGTAGGTGAAG-3′; *GAPDH* Forward: 5′-AGCCTCAAGATCATCAGCAATGCC-3′, Reverse: 5′-TGTGGTCATGAGTCCTTCCACGAT-3′.

## Results

### EGCG and luteolin synergistically inhibit TGF-β-induced fibronectin expression

**Figure 1:**
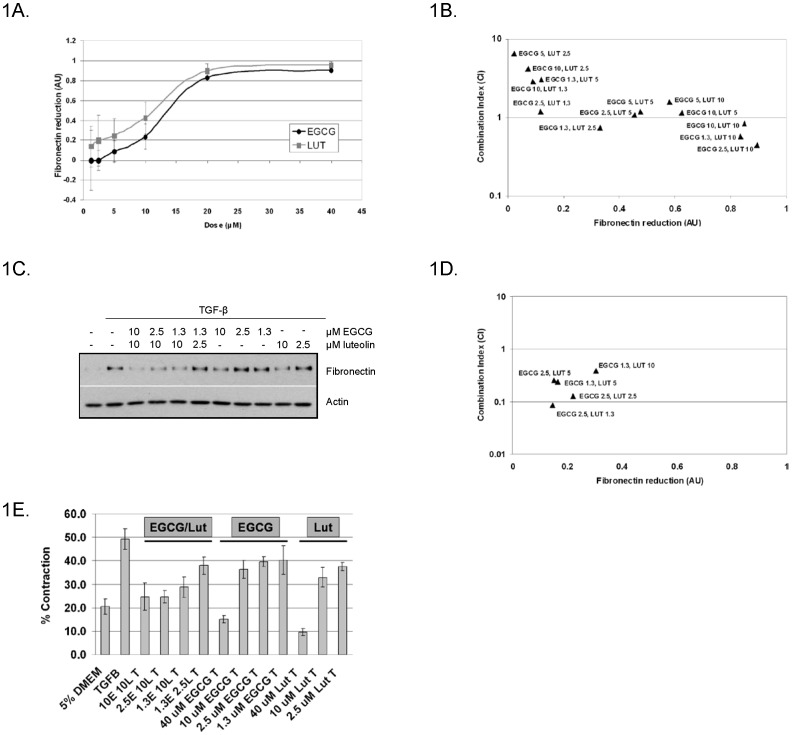
Combinations of EGCG and luteolin synergistically inhibit TGF-β-induced fibronectin expression and reduce fibroblast matrix contraction. (A–E) WPMY-1 and HPS-19I cells were treated with or without 5 ng/ml TGF-β in the presence of EGCG and/or luteolin at the indicated concentrations for 24 hours. Cell lysates were analyzed by western blot and densitometry was used to determine reduction of fibronectin expression. (A) Dose response data are expressed as mean ± S.E.M.; n = 3. (B) WPMY-1 synergy was determined using CompuSyn software, synergy = CI<1; n = 3. (C) Representative western blot of synergistic combinations of EGCG and luteolin with corresponding single agent doses. (D) HPS-19I synergy was determined using CompuSyn software, synergy = CI<1; n = 3. (E) WPMY-1 cells were treated with or without 5 ng/ml TGF-β in the presence of EGCG and/or luteolin at the indicated concentrations for 4 days post seeding. Data are shown as percent contracted area; n = 3.

The WPMY-1 fibroblast cell line was derived from a non-malignant region adjacent to the prostate and expresses markers of myofibroblasts, including fibronectin and α-SMA [32]. Upon differentiation, myofibroblasts secrete the ECM proteins collagen and fibronectin. Cytokines and growth factors can induce and enhance this phenotype, further augmenting cancer progression. Using TGF-β as an enhancer of the myofibroblast phenotype [33], we investigated whether EGCG and luteolin were able to prevent further induction of the myofibroblast phenotype. WPMY-1 cells were treated for 24 hours with TGF-β with a range of concentrations of EGCG or luteolin to generate dose response curves showing effects on TGF-β-induced fibronectin expression. Representative images are shown in Fig. S1 and images from three independent experiments were used to quantitate the effects of EGCG and luteolin as shown in Fig. 1A. Fig. S2A demonstrates that 20–40 µM EGCG inhibited TGF-β-induced α-SMA and collagen expression, while lower concentrations did not antagonize TGF-β-mediated effects significantly. Luteolin dose dependently inhibited α-SMA (Fig. S2B) and fibronectin induction with a near-complete block observed at 20 µM (Fig. 1A and Fig. S1B). When used in combination, EGCG and luteolin were found to be more efficacious at reducing fibronectin induction at concentrations that proved to be less effective when used alone. Fig. 1B shows the combinatorial effects of various concentrations of EGCG and luteolin. Four specific combinations were found to be synergistic as shown by having a CI <1. A representative western blot containing these synergistic combinations and their single agent doses is shown in Fig. 1C. Using another prostate fibroblast cell line, HPS-19I, we found that EGCG and luteolin were not as effective at reducing fibronectin; however, the same combined concentration range resulted in multiple synergistic combinations (Fig. 1D). Overall, these data suggest that EGCG and luteolin can be used in combination at sub-optimal single agent concentrations to reduce fibronectin expression.

### EGCG and luteolin reduce TGF-β-induced ECM contraction

Fibroblasts, when plated in a 3-dimensional matrix, stimulated with an inducer of differentiation, and released from the tissue culture plastic produce a contractile force on the matrix, a defining function of myofibroblasts [31]. In order to determine if EGCG and luteolin inhibited myofibroblast functionality, 3-dimensional ECM remodeling assays were performed. Synergistic combinations of EGCG and luteolin, as determined in Fig. 1B, had slightly better or at least equal effects on preventing matrix contraction compared to single agents alone (Fig. 1E). Therefore, combinations of EGCG and luteolin that synergistically reduce fibronectin expression are also effective at reducing myofibroblast contractility.

### EGCG and luteolin do not synergistically reduce WPMY-1 fibroblast cell growth and viability

**Figure 2:**
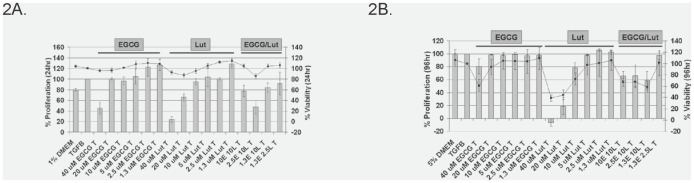
EGCG and luteolin do not synergistically reduce WPMY-1 cell proliferation and viability. WPMY-1 cells were treated with EGCG and/or luteolin at the indicated concentrations for 24 hours in 1% FBS DMEM (A) or 96 hours in 5% FBS DMEM (B). Proliferation (indicated by bars; left y-axis) and metabolically viable cells (indicated by line; right y-axis) are shown as mean percent confluency or viability ± S.E.M. normalized to TGF-β; 1%: n = 2, 5%: n = 3.

EGCG and luteolin have been shown previously to inhibit cell growth in a variety of cell types [20], [34], [35]. In order to determine if these polyphenols disrupt the myofibroblast phenotype by affecting cell growth, proliferation assays were performed on WPMY-1 cells over 24 hours in 1% FBS DMEM or 96 hours in 5% FBS DMEM to mimic conditions used in cell signaling and ECM remodeling assays, respectively. Compared to TGF-β-treated cells, when used at the highest concentrations (40 µM) EGCG and luteolin inhibited cell proliferation by approximately 50% and 80% at 24 hours posttreatment and 20% and 100% at 96 hours posttreatment, respectively (bars, Fig. 2A and 2B). Viability assays showed that EGCG and luteolin had no effect on the metabolic capacity of the cells present at the end of the proliferation assays at 24 hours posttreatment, and at 96 hours posttreatment only higher concentrations of the compounds decreased viability (line, Fig. 2A and 2B). A comparison of combinations to single agent treatments revealed that combinations did not affect proliferation or viability any greater than single agent treatments. This information suggests that the effects of luteolin and EGCG on the myofibroblast phenotype are not the result of changes to cell viability or proliferation.

### EGCG and luteolin inhibit TGF-β-induced downstream signaling

The primary signaling pathway mediating TGF-β signaling involves activation of the Smad signaling proteins [36]. Additional pathways involved in myofibroblast phenotypes include the PTK2 (FAK)/AKT1/2 (AKT)/PIK3CA (PI-3K) and the MAPK1 (ERK) signaling pathways [37]. To determine if these pathways are targeted by EGCG and luteolin, WPMY-1 myofibroblasts were treated with TGF-β +/− EGCG or luteolin overnight. As indicated by western blot analysis in Fig. S3, TGF-β treatment activated the Smad and, to a lesser extent, FAK pathways as noted by increased phosphorylation. EGCG and luteolin at 40 µM reduced phosphorylation of Smad2 and FAK (Fig. S3A and S3B). It has been reported that EGCG is able to inhibit TGF-β receptor expression [38]; however, in our system, EGCG nor luteolin decreased TGF-β receptor levels (Fig. S3).

**Figure 3:**
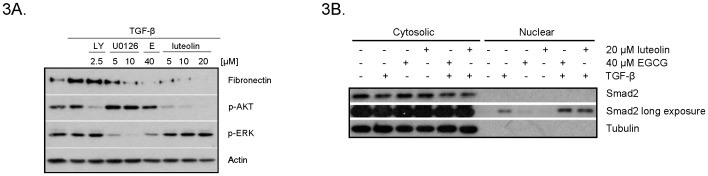
EGCG may block fibronectin induction through inhibition of ERK. (A) WPMY-1 cells were treated with or without 5 ng/ml TGF-β for 24 hours and PI-3K inhibitor LY294002 or ERK inhibitor U0126, EGCG or luteolin at the indicated concentrations. Cell lysates were analyzed by western blot. (B) WPMY-1 cells were treated with or without 5 ng/ml TGF-β in the presence of 20 µM luteolin or 40 µM EGCG for 1 hour prior to nuclear fractionation. Western blot was used to analyze 5 µg of protein per sample from each fraction.

Fig. 3A reveals EGCG diminished phosphorylated ERK while luteolin decreased phosphorylated AKT. In order to determine if EGCG and luteolin block TGF-β-induced fibronectin expression through inhibition of signaling through AKT or ERK, WPMY-1 fibroblasts were treated with TGF-β +/− the PI-3K inhibitor LY294002 or the ERK inhibitor U0126. We found that U0126, but not LY294002, reduced TGF-β-induced fibronectin induction, suggesting that the ERK pathway is necessary for this process. Based on these data, it is likely that EGCG may inhibit fibronectin induction through targeting the ERK pathway.

### EGCG and luteolin do not inhibit Smad2 nuclear translocation

To further investigate the mechanisms by which EGCG and luteolin inhibit TGF-β-induced signaling, we examined potential effects on Smad nuclear translocation. Upon TGF-β stimulation, Smad proteins become phosphorylated and translocate into the nucleus to activate TGF-β responsive genes [39]. The importance of Smad activity in regards to myofibroblast phenotypes is somewhat controversial. In some experimental systems, Smad2 has been reported to be necessary for α-SMA induction [40]; however, inhibition of myofibroblast differentiation has occurred without inhibition of Smad2 phosphorylation or translocation [41]. While it appears that EGCG inhibits Smad2 phosphorylation at 40 µM (Fig. S3), luteolin did not inhibit Smad2 activity at concentrations which inhibited TGF-β-induced differentiation (Fig. 1A and Fig. S2 and S3). Regardless, in order to assess if EGCG and luteolin were capable of inhibiting Smad2 nuclear translocation, WPMY-1 cells were treated with TGF-β +/− EGCG or luteolin and nuclear fractionation was performed. Fig. 3B demonstrates that TGF-β treatment stimulated Smad2 nuclear translocation. However, neither EGCG nor luteolin were capable of inhibiting TGF-β-induced Smad2 nuclear translocation at concentrations that block fibronectin production. These data suggest that TGF-β-induced myofibroblast fibronectin induction is independent of Smad2 nuclear translocation in our system.

### WPMY-1 fibronectin induction is not dependent on lipid raft integrity

**Figure 4:**
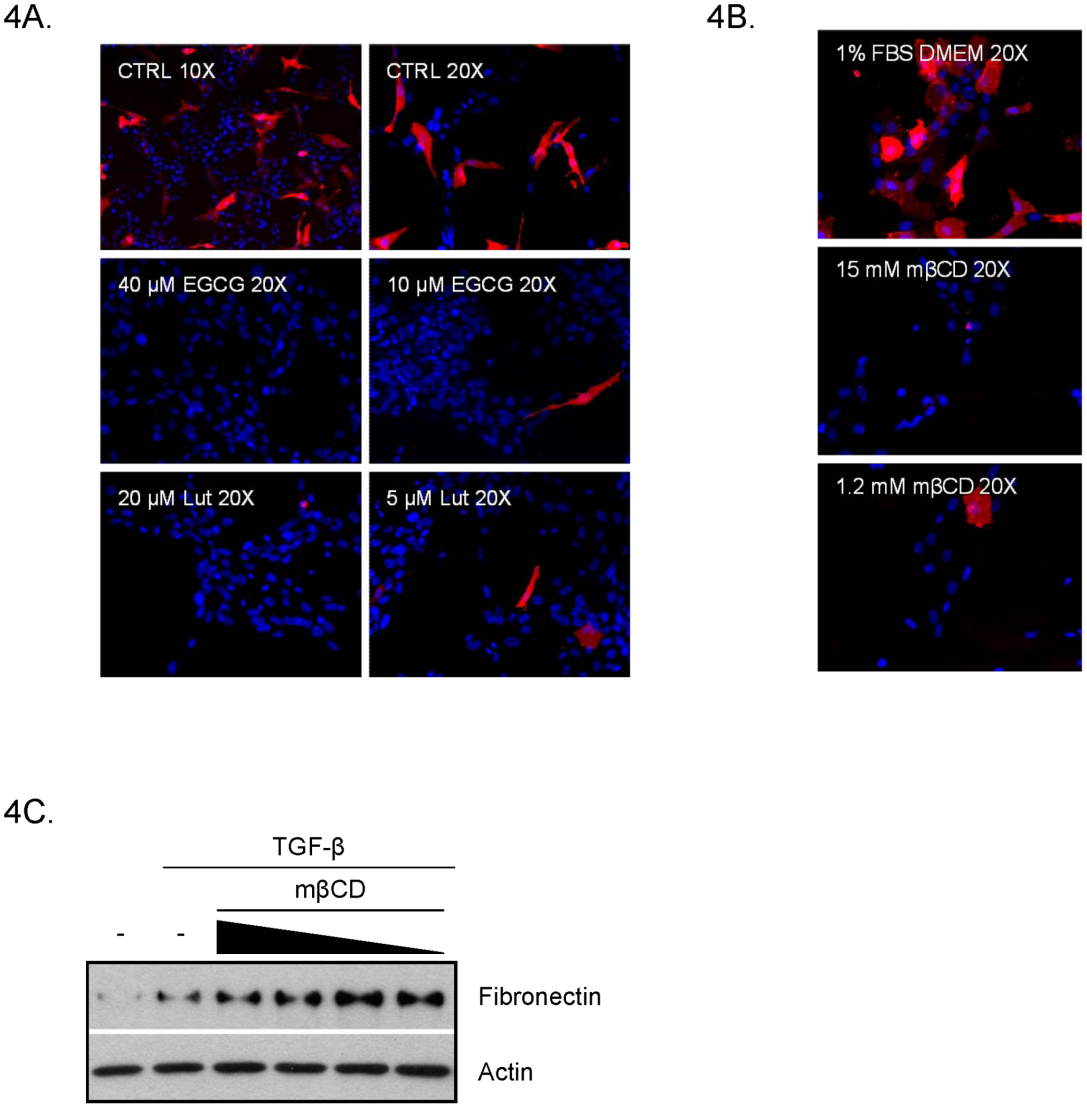
Fibronectin induction is not dependent on lipid raft integrity. (A) WPMY-1 cells were treated with or without 5 ng/ml TGF-β in the presence of 10 or 40 µM EGCG or 5 or 20 µM luteolin for 24 hours. (B) Cells were treated with the indicated concentrations of mβCD for 30 minutes. (A and B) Cells were stained with 1 µM DiIC16 (red) and DAPI (blue). Representative images are shown. (C) Cells were treated with 1.2–15 mM mβCD for 30 minutes, washed, then treated overnight with TGF-β. Indicated proteins were analyzed by western blot.

It has become widely accepted that the dynamic signaling of the TGF-β pathway is responsive to cholesterol levels [42]. In general, the TGF-β receptor complexes are believed to be localized primarily in non-lipid raft domains. Upon stimulation, the receptors become internalized via clathrin-mediated endocytosis and signaling is continued in endosomes. Alternatively, TGF-β receptors can move into lipid raft compartments where they become internalized via caveolin-1-mediated pathways and undergo degradation [42], [43]. Reports suggest that EGCG is capable of targeting multiple signaling pathways through alterations in membrane fluidity [26], [44] and luteolin is known to affect lipogenesis [45]. In order to determine if EGCG and luteolin affect TGF-β signaling via alterations in membrane fluidity, WPMY-1 fibroblasts were treated with EGCG or luteolin overnight and stained with DiIC16 which preferentially stains ordered lipid domains (lipid rafts). As shown in Fig. 4A, immunofluorescence microscopy reveals that DiIC16 readily stained control cells; however, EGCG and luteolin dose-dependently inhibited DiIC16 membrane incorporation. These data demonstrate that concentrations of EGCG of luteolin that reduce the myofibroblast phenotype also disrupt lipid rafts, suggesting that these compounds may work to inhibit myofibroblast-mediated effects through alteration of lipid composition.

To further determine the role of lipid raft integrity in fibronectin induction, WPMY-1 cells were treated with TGF-β +/− mβCD to remove cholesterol from cells. Fig. 4B shows that mβCD disrupts lipid rafts even at the lowest concentration used; however, treatment of WPMY-1 cells with mβCD did not inhibit fibronectin production induced by TGF-β (Fig. 4C). Therefore, although EGCG and luteolin are capable of disrupting lipid rafts, in our system, fibronectin induction by TGF-β is independent of lipid raft integrity.

### EGCG and luteolin inhibit RhoA activation

RhoA is an important mediator of downstream TGF-β signaling and has been found to be involved in fibronectin induction in multiple cell lines and stimulatory conditions [46], [47]. RhoA is a member of the Rho family of GTPases and shares homology with 2 isoforms, RhoB and RhoC. Rho GTPases regulate cytoskeleton dynamics and cell movement. Activity of Rho GTPases depends on dynamic cycling between inactive Rho-GDP and active Rho-GTP in response to various stimuli [48]. Following activation, RhoA is translocated to the cell membrane where it is geranylgeranylated [49]. These steps are required for downstream effects of RhoA signaling. Because RhoA is known to participate in myofibroblast phenotypes, we sought to determine if EGCG and luteolin prevent TGF-β-mediated effects by targeting RhoA.

**Figure 5:**
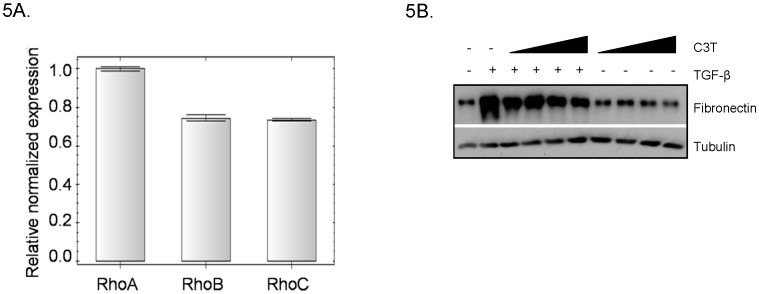
Rho activity is required for WPMY-1 fibronectin induction. (A) qRT-PCR was performed, following RNA isolation and cDNA synthesis, on WPMY-1 cells under normal culture conditions. Expression of the indicated genes was analyzed; n = 2. (B) WPMY-1 cells were pre-treated with the Rho A/B/C inhibitor C3 Transferase at 1.2–10 µM for 8 hours prior to addition of 5 ng/ml TGF-β for 24 hours. Cell lysates were analyzed by western blot.

Data from qRT-PCR analysis show that WPMY-1 cells express all 3 isoforms of *RHO* (Fig. 5A). In order to determine if Rho signaling is important in TGF-β-induced fibronectin expression in prostate myofibroblasts, WPMY-1 cells were treated with TGF-β +/− the general Rho inhibitor, C3 transferase. C3 transferase is an ADP-ribosylase that modifies Rho at the GTP binding site, preventing Rho activation. Fig. 5B demonstrates that C3 transferase inhibited TGF-β-induced fibronectin production in WPMY-1 cells suggesting that Rho activity is an important mediator of fibronectin expression.

To directly study RhoA, we stably reduced RhoA expression using lentiviral-delivered shRNA in WPMY-1 cells (Fig. S4A). Reduction in levels of RhoA did not affect expression of RhoB or RhoC (Fig. S4B) but did decrease expression of the myofibroblast markers fibronectin (*FN1)* and to a lesser extent collagen (*COL1A1)* and α-SMA (*ACTA2*) (Fig. S4C). Collectively, these data implicate a role for RhoA in WPMY-1 myofibroblast phenotypes.

**Figure 6:**
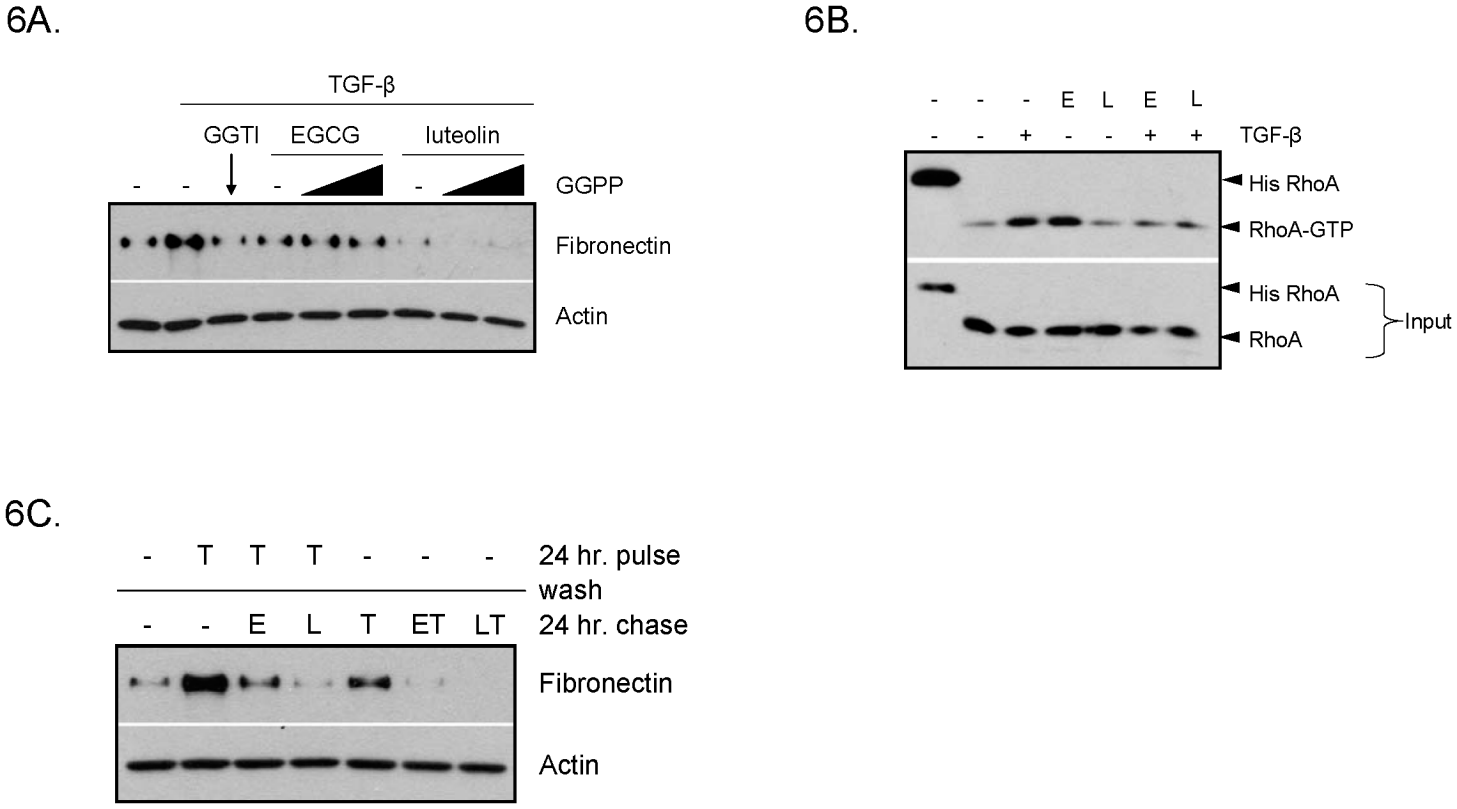
EGCG and luteolin reduce RhoA activation and reverse TGF-β-induced fibronectin expression. (A) WPMY-1 cells were pre-treated 8 hours with 20 µM GGTI-2133, 20 µM EGCG or 20 µM luteolin in the presence or absence of 10 µM or 40 µM geranylgeranylpyrophosphate (GGPP) prior to co-treatment with TGF-β for an additional 24 hours. Western blot was used to determine levels of the indicated proteins. (B) WPMY-1 cells were pre-treated 30 minutes in 1% FBS DMEM with EGCG (E) or luteolin (L) prior to addition of TGF-β for an additional 15 minutes. Rhotekin-RBD beads were used to pulldown active RhoA. (C) WPMY-1 cells were pulsed with TGF-β for 24 hours, washed with PBS, then chased with 40 µM EGCG or luteolin with or without TGF-β for an additional 24 hours.

Since EGCG and luteolin affected cholesterol-dependent lipid rafts, we questioned whether EGCG and luteolin could affect geranylgeranylation of RhoA by obstructing cholesterol synthesis. Geranylgeranyl pyrophosphate is a product of the cholesterol synthesis pathway. Accordingly, one might predict that EGCG and luteolin are able to prevent geranylgeranylation of RhoA through inhibition of this pathway. To investigate this we used a geranylgeranyl transferase inhibitor, GGTI-2133, in an attempt to mimic the effects of EGCG and luteolin on fibronectin induction. General inhibition of geranylgeranylation prevented fibronectin induction supporting the role for RhoA geranylgeranylation in this process. However, when WPMY-1 cells were treated with luteolin or EGCG in the presence of exogenous geranylgeranylpyrophosphate (GGPP), fibronectin expression was not rescued (Fig. 6A). Based on these data, we concluded that EGCG and luteolin do not inhibit fibronectin induction through inhibition of RhoA geranylgeranylation by depletion of geranylgeranyl pyrophosphate.

To further investigate how RhoA could be involved in fibronectin induction, WPMY-1 fibroblasts were treated with TGF-β +/− EGCG or luteolin and levels of activated RhoA (RhoA-GTP) were measured. As expected, TGF-β treatment increased RhoA activation, whereas EGCG and luteolin reduced the induction of RhoA-GTP in the presence of TGF-β (Fig. 6B). We consistently noticed that EGCG alone activated RhoA similar to TGF-β, yet EGCG and TGF-β together resulted in basal, unstimulated levels of activated RhoA (Fig. 6B).

### EGCG and luteolin reverse TGF-β-induced fibronectin expression

In order to determine if EGCG and luteolin reduced TGF-β-induced fibronectin expression predominantly through inhibition of early signaling events, such as RhoA activation, we pre-treated WPMY-1 cells with TGF-β followed by EGCG and luteolin. We found that EGCG and luteolin were able to reverse fibronectin expression in cells that were already induced with TGF-β (Fig. 6C). This indicated that EGCG and luteolin are able to reduce fibronectin expression in a manner independent of their effects on early signaling events.

## Discussion

Our laboratory and others have found that EGCG and luteolin are capable of preventing development and/or maintenance of myofibroblast phenotypes when used at relatively high micromolar concentrations. Our most effective dose of EGCG was similar to that found by Kang *et al.* who showed that approximately 20–40 µM EGCG inhibited α-SMA and collagen type I production in nasal polyp-derived fibroblasts [50] and to another study which found that 40 µM EGCG largely obliterated fibronectin production in primary dermal fibroblasts [51]. However, these concentrations are likely to have off-target effects, causing deleterious toxicity *in vivo* and likely represent concentrations that cannot be achieved *in vivo*. Therefore, we sought to determine if EGCG and luteolin could be effective at lower concentrations in combination. As an example, we found that luteolin at 40 µM inhibits fibronectin expression by 90% but was only 50% effective at 10 µM. Luteolin at 10 µM in combination with EGCG at 10, 2.5, or 1.3 µM reduces fibronectin by ∼90% and the inhibition of proliferation changes from 80% with 40 µM luteolin to only 10–50% with 10 µM luteolin in combination with EGCG. In other words, the use of luteolin and EGCG in combination demonstrates similar efficacy and less toxicity than higher concentrations of either compound alone. These data also suggest that since these cells do not grow much in a 24 hour period in 1% FBS DMEM (Fig. S5), it is unlikely that EGCG and luteolin reduce fibronectin levels through effects on proliferation.

Notably, mechanical force can enhance tumor cell invasion [52]; therefore, the ability of myofibroblasts to induce ECM contraction is a potential target for reducing tumor cell invasion and metastasis. In ECM remodeling assays, we found that EGCG and luteolin not only reduced TGF-β-induced collagen matrix contraction but also reduced contraction beyond basal levels at high (40 µM) concentrations. This could be due to inhibition of autocrine TGF-β signaling which is necessary to maintain a myofibroblast phenotype [40]. Or, because a significant change in the growth of cells occurs over 96 hours (Fig. S5), the effects of high concentrations of EGCG and luteolin on contraction may be attributed to slower proliferation rates. However, lower concentrations can specifically prevent myofibroblast phenotypic changes in a manner not attributable to reduced viability. By combining lower doses of EGCG and luteolin, we were able to reduce TGF-β-induced fibronectin induction and collagen matrix contraction to similar levels seen with higher doses of EGCG or luteolin alone. Collectively, these data demonstrate that combinations of lower, more physiologically obtainable doses of EGCG and luteolin can be equally effective, if not more effective, at preventing myofibroblast-mediated effects than higher concentrations of either compound alone while also reducing potential toxicities.

Mechanistically, we found that EGCG inhibits the MAPK pathway, while luteolin targets the PI-3K pathway in fibroblasts, but only the ERK pathway seems to be necessary for TGF-β-induced fibronectin expression. Our data showing that RhoA knockdown (KD) decreased fibronectin expression are similar to the results reported by Peng *et al.* who found that RhoA siRNA reduced glucose induction of fibronectin in rat mesangial cells [53]. EGCG and luteolin mimicked the reported ability of Simvastatin, a cholesterol synthesis inhibitor, to reduce fibronectin levels [49]; however, our data indicate that luteolin and EGCG prevent fibronectin induction through inhibition of RhoA activation, while Simvastatin works through inhibition of RhoA geranylgeranylation downstream of GTP binding [49]. This is supported by the inability of exogenous geranylgeranylpyrophosphate to rescue fibronectin induction blocked by EGCG and luteolin, because excess free geranylgeranyl would not be able to modify RhoA in the absence of RhoA-GTP. Therefore, EGCG and luteolin appear to target different arms of the TGF-β signaling axis, but also share RhoA activation as a common target for preventing fibronectin induction.

Given that promotion of reactive stroma occurs during early stages of cancer progression [54], the finding that EGCG and luteolin were able to reverse the already initiated cascade toward a cancer-promoting myofibroblast phenotype is exceptionally important. Based on numerous reports, it is clear that these polyphenols are able to act on multiple molecular targets. Our findings support this idea and demonstrate that EGCG and luteolin are able to work at multiple points along the TGF-β signaling cascade to not only block, but also, reverse the hyperactive myofibroblast phenotype. We have shown that these compounds inhibit early signaling events required for TGF-β-induced fibronectin induction, including activation of RhoA and ERK; however, the ability of EGCG and luteolin to reverse fibronectin expression already induced by TGF-β suggests that these compounds may also be able to disrupt a previously-fired signaling circuit that is required for maintaining the myofibroblast phenotype, such as the YAP pathway [31].

Our data provide the first evidence that EGCG and luteolin can be used in combination at low doses to prevent myofibroblast-mediated effects, particularly fibronectin induction and ECM remodeling. These data in combination with our previous reports [24], [27] and others [55] provide further evidence that these natural compounds have clinical potential for not only targeting tumor epithelial cells, but also for targeting the surrounding tumor microenvironment. Furthermore, an earlier study supported that green tea catechin consumption consisting, in part, of EGCG could slow progression from high grade prostatic intraepithelial neoplasia (HGPIN) to prostate cancer [56]. Combinations of EGCG and luteolin could prove to be even more effective than green tea catechin treatments alone.

We are aware of issues with bioavailability of naturally occurring dietary compounds; however, others have begun testing nanoparticle delivery of luteolin and EGCG and found increased delivery and significant efficacy of these compounds [23], [57]. The evidence that EGCG and luteolin are able to target both the tumor itself and the tumor microenvironment suggests that nanoparticles targeting both sites may prove to be especially beneficial. Although beyond the scope of this study, it would be interesting to target each site separately, in order to determine which tissue site is the more effective target for reducing tumor progression. Additionally, our data suggesting that EGCG and luteolin synergistically reduce fibronectin expression imply that it may be possible to achieve biologically active concentrations when combining EGCG and luteolin that may not be possible when using either compound alone.

The data presented herein showing that EGCG and luteolin target myofibroblasts help to explain reduced levels of stroma-derived cytokines and growth factors detected in prostate cancer patients treated with Polyphenon E [27]. Notably, these data have implications for therapeutic intervention in diseases beyond cancer, such as fibrosis and renal disease, that arise due to aberrations in fibronectin expression [46], . Particularly, the finding that EGCG and luteolin are able to reverse fibronectin induction has significant implications in potentially reversing already prevalent disease driven by fibronectin expression.

In conclusion, we have provided evidence that combinations of EGCG and luteolin allow less toxic concentrations of these compounds to be used while maintaining effectiveness in restricting the cancer-promoting myofibroblast phenotype. Mechanistically, these natural dietary agents prevent further enhancement of myofibroblast-mediated effects through inhibition of signaling cascades downstream of TGF-β. Therefore, combinations of EGCG and luteolin could be beneficial by not only directly targeting tumor epithelial cells, but also preventing and reversing advancement of the myofibroblast phenotype. Long-term, low dose dual inhibition of both the tumor epithelia and the tumor microenvironment using EGCG and luteolin could prove to be efficacious in preventing formation of advanced stage cancer.

## Supporting Information

Figure S1 **EGCG and luteolin reduce TGF-β-induced fibronectin expression.** WPMY-1 cells were treated with or without 5 ng/ml TGF-β in the presence of EGCG (A) or luteolin (B) at the indicated concentrations for 24 hours. Cell lysates were analyzed by western blot.
(TIF)

Figure S2 **EGCG inhibits TGF-β induction of myofibroblast markers.** WPMY-1 cells were treated with or without 5 ng/ml TGF-β in the presence of EGCG (A) or luteolin (B) at the indicated concentrations for 24 hours. Cell lysates were analyzed using western blot.
(TIF)

Figure S3 **EGCG and luteolin reduce Smad2 and FAK signaling at high concentrations.** WPMY-1 cells were treated in the presence or absence of 5 ng/ml TGF-β with the indicated concentrations of EGCG (A) or luteolin (B) for 24 hours. Western blot was used to analyze activation and expression of the indicated proteins.
(TIF)

Figure S4 **RhoA KD reduces expression of myofibroblast markers. (**A) WPMY-1 cells were treated with lentivirus expressing four distinct shRNA sequences (i–iv) targeted to RhoA. (B, C) RNA was isolated from WPMY-1 NT and RhoA KD (iii, iv) cells. Expression of the indicated genes was analyzed by qRT-PCR; n = 2.
(TIF)

Figure S5 **WPMY-1 proliferation rates vary with time and growth conditions.** WPMY-1 proliferation was analyzed for 24 hours in 1% FBS DMEM or 96 hours in 5% FBS DMEM. Data are shown as mean percent confluency ± S.E.M.; 1%: n = 2, 5%: n = 3.
(TIF)
