# Supplementary figures and images for: The Polyphenols (−)-Epigallocatechin-3-Gallate and Luteolin Synergistically Inhibit TGF-β-Induced Myofibroblast Phenotypes through RhoA and ERK Inhibition
Source: PLoS One. 2014 Oct 1;9(10):e109208. doi: 10.1371/journal.pone.0109208 (PMC4182889; doi:10.1371/journal.pone.0109208)

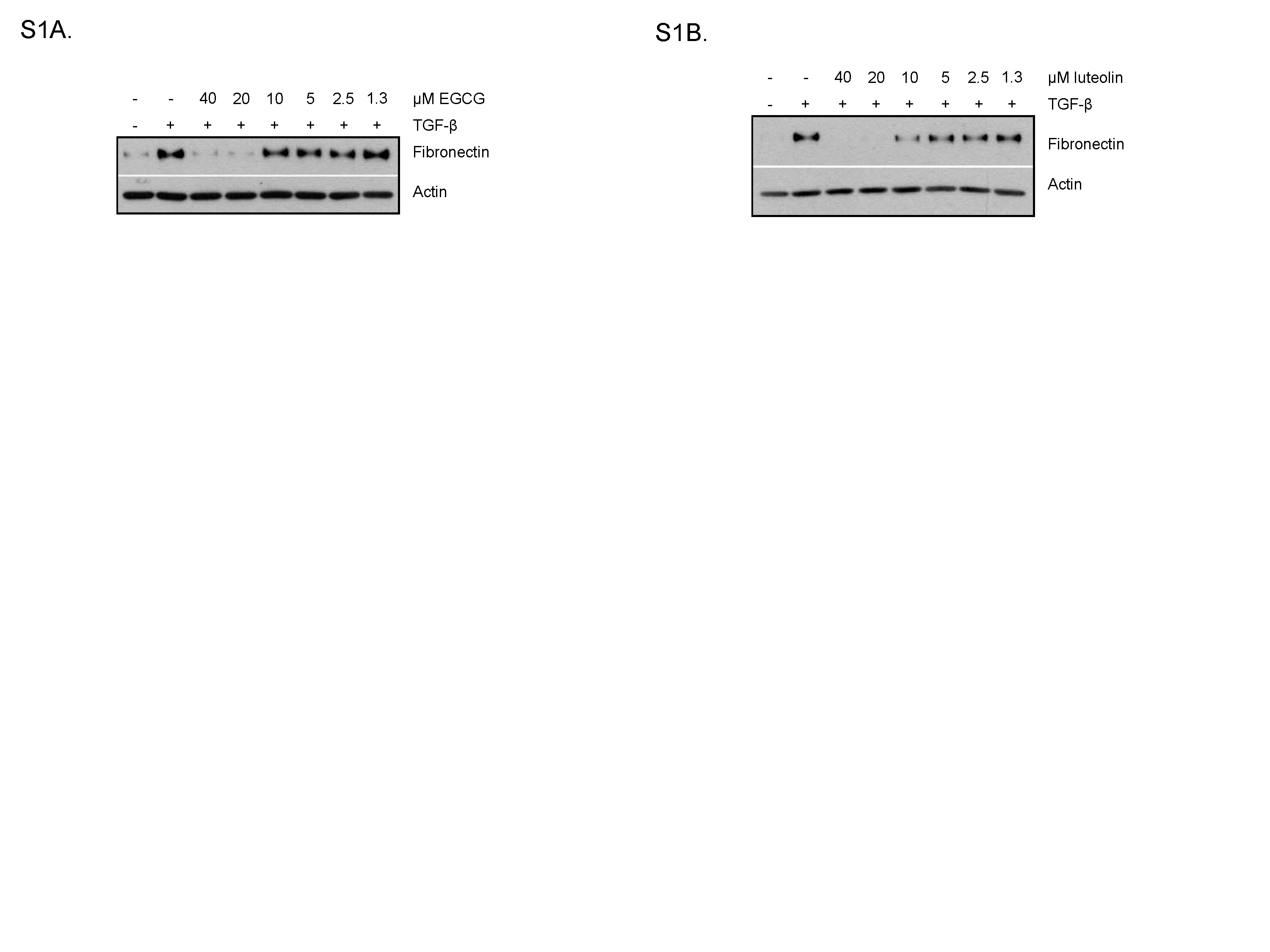

Supplement: Figure S1 — EGCG and luteolin reduce TGF-β-induced fibronectin expression. WPMY-1 cells were treated with or without 5 ng/ml TGF-β in the presence of EGCG (A) or luteolin (B) at the indicated concentrations for 24 hours. Cell lysates were analyzed by western blot. (TIF) [file pone.0109208.s001.tif]

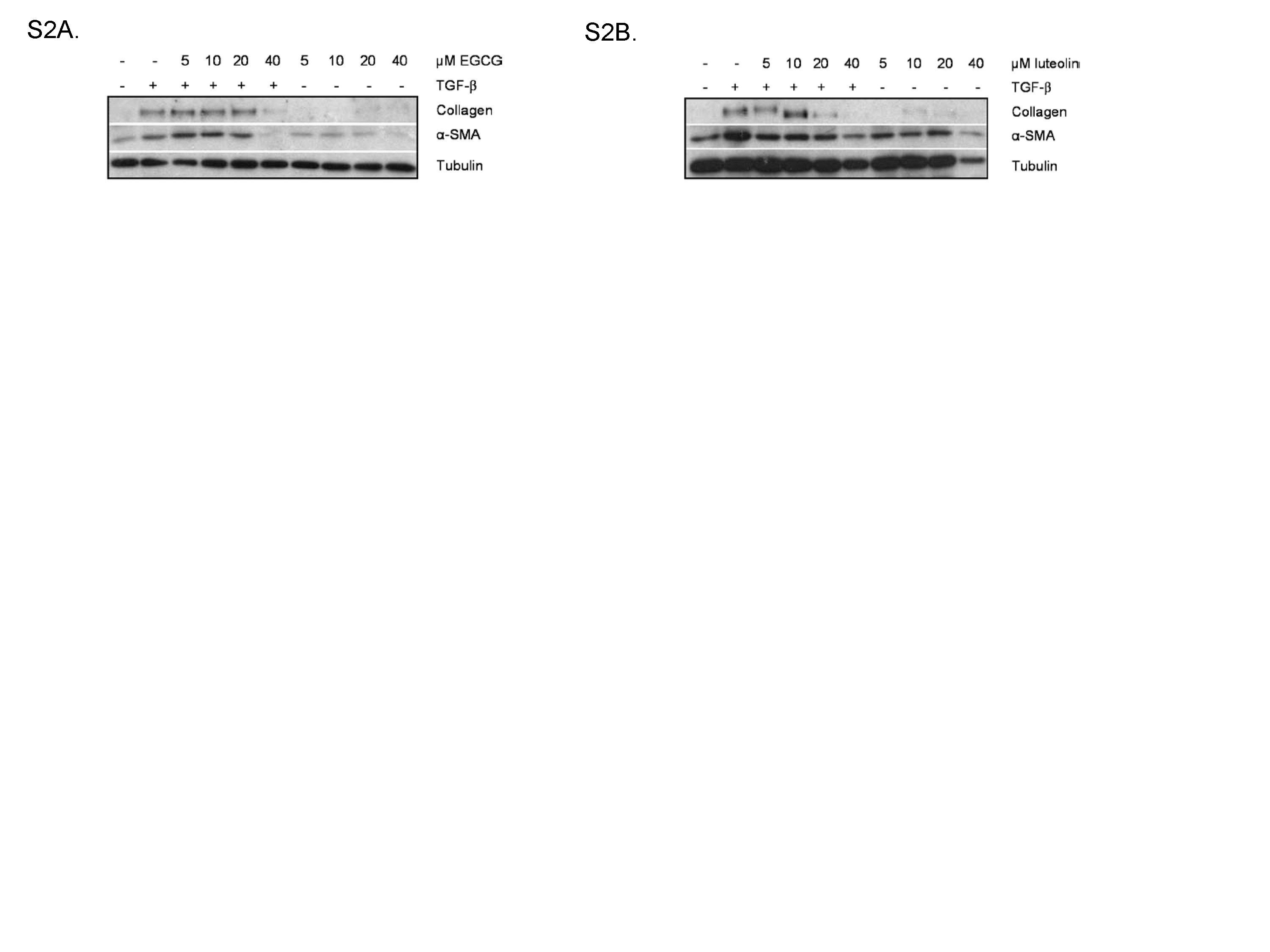

Supplement: Figure S2 — EGCG inhibits TGF-β induction of myofibroblast markers. WPMY-1 cells were treated with or without 5 ng/ml TGF-β in the presence of EGCG (A) or luteolin (B) at the indicated concentrations for 24 hours. Cell lysates were analyzed using western blot. (TIF) [file pone.0109208.s002.tif]

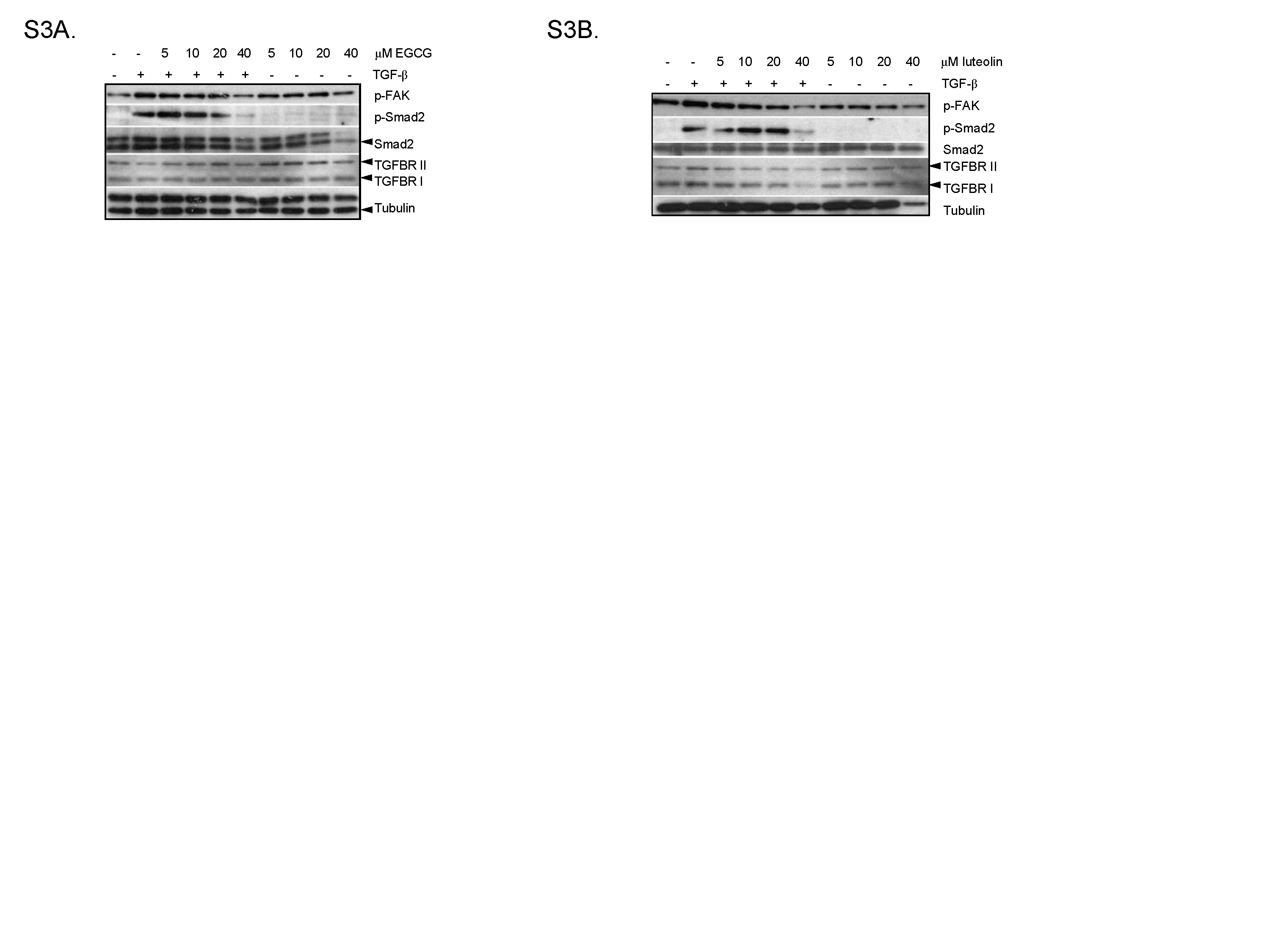

Supplement: Figure S3 — EGCG and luteolin reduce Smad2 and FAK signaling at high concentrations. WPMY-1 cells were treated in the presence or absence of 5 ng/ml TGF-β with the indicated concentrations of EGCG (A) or luteolin (B) for 24 hours. Western blot was used to analyze activation and expression of the indicated proteins. (TIF) [file pone.0109208.s003.tif]

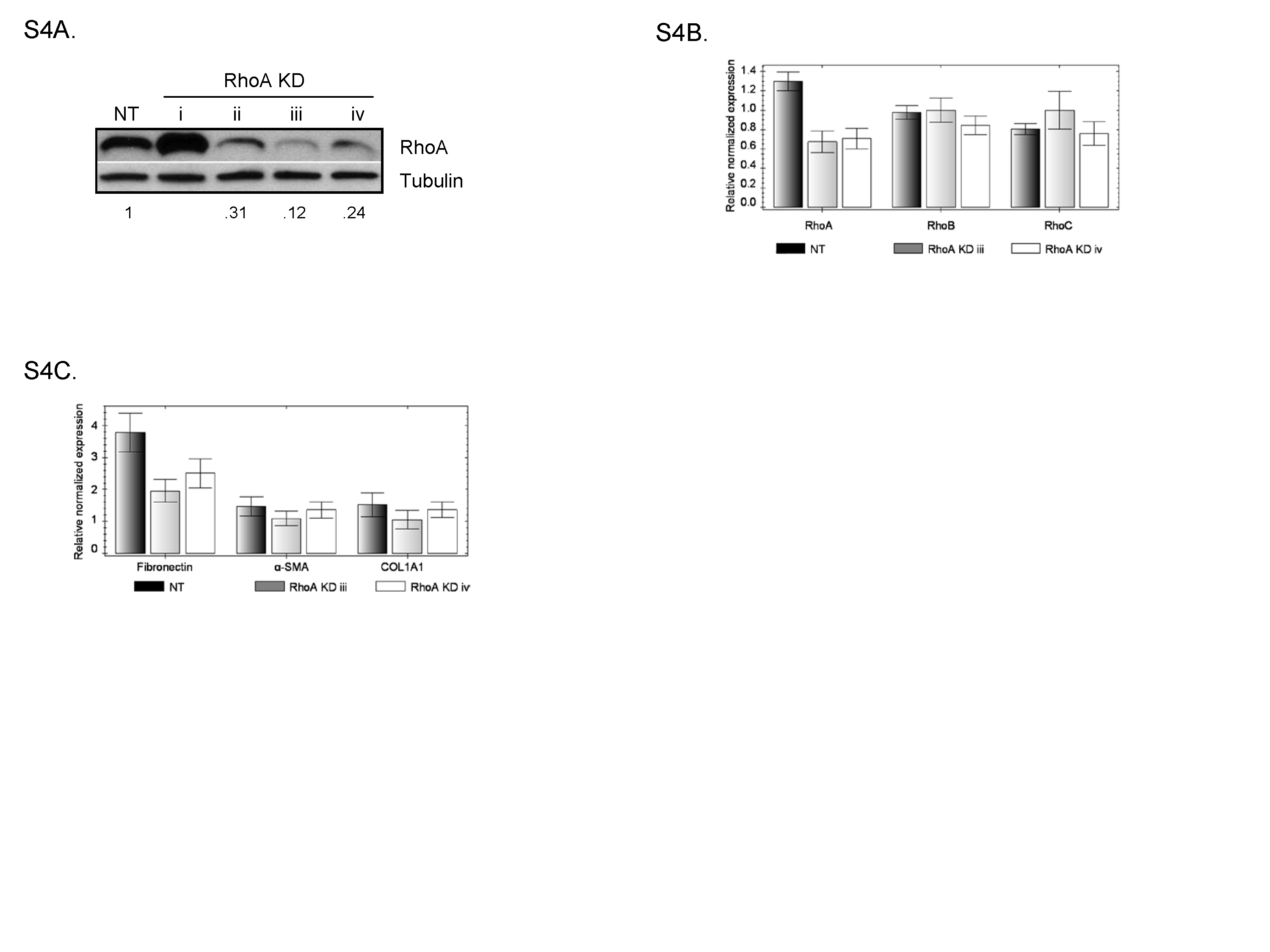

Supplement: Figure S4 — RhoA KD reduces expression of myofibroblast markers. (A) WPMY-1 cells were treated with lentivirus expressing four distinct shRNA sequences (i–iv) targeted to RhoA. (B, C) RNA was isolated from WPMY-1 NT and RhoA KD (iii, iv) cells. Expression of the indicated genes was analyzed by qRT-PCR; n = 2. (TIF) [file pone.0109208.s004.tif]

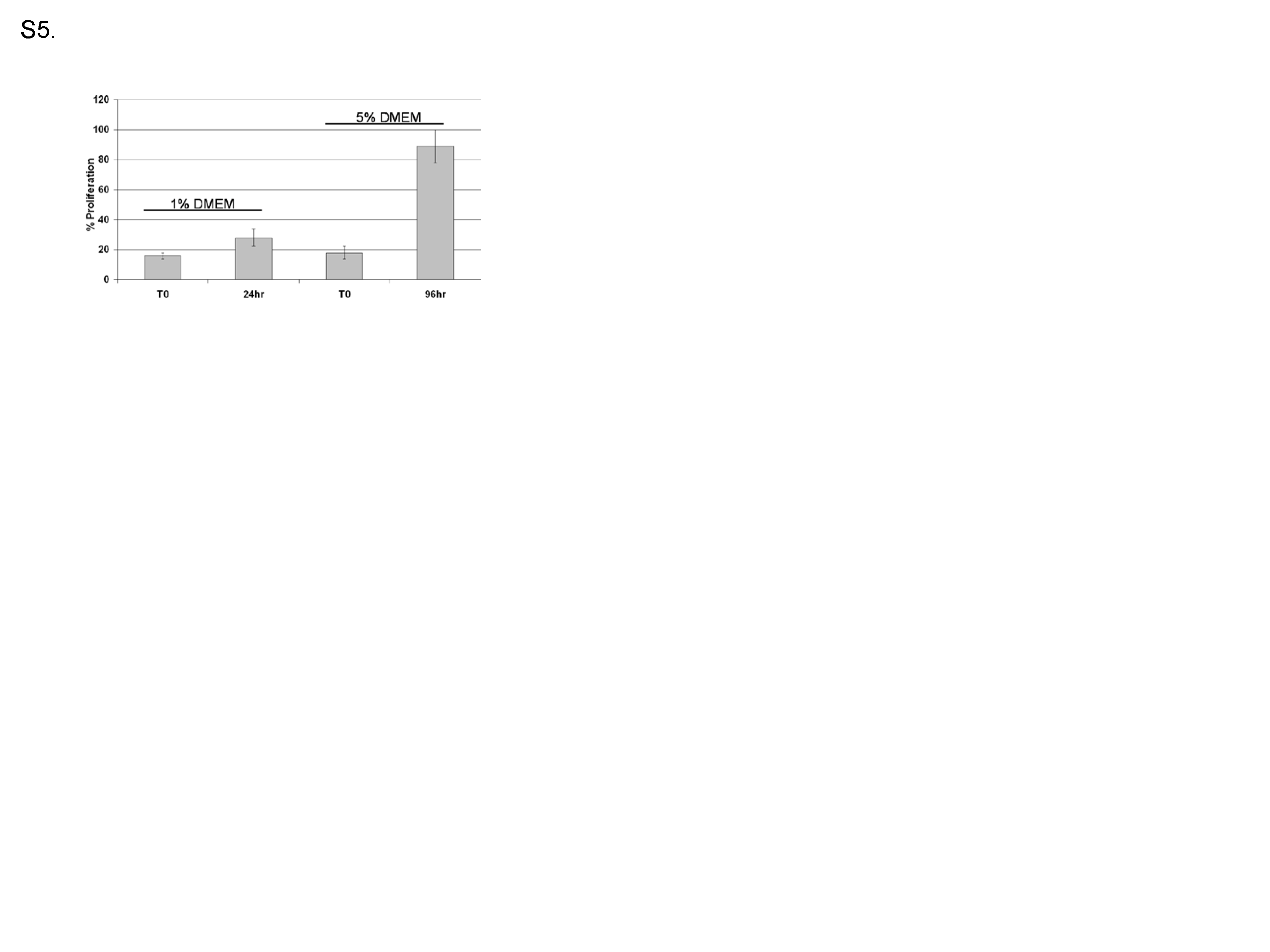

Supplement: Figure S5 — WPMY-1 proliferation rates vary with time and growth conditions. WPMY-1 proliferation was analyzed for 24 hours in 1% FBS DMEM or 96 hours in 5% FBS DMEM. Data are shown as mean percent confluency ± S.E.M.; 1%: n = 2, 5%: n = 3. (TIF) [file pone.0109208.s005.tif]
